# Implementation of the PulsePoint smartphone application for crowd-sourcing bystander resuscitation

**DOI:** 10.1186/cc13674

**Published:** 2014-03-17

**Authors:** SC Brooks, H Worthington, T Gonedalles, B Bobrow, LJ Morrison

**Affiliations:** 1 Queen's University, Kingston, Canada; 2 St Michael's, Toronto, Canada; 3 University of Arizona Medical College, Phoenix, AZ, USA; 4 University of Toronto, Canada

## Introduction

Only a minority of patients suffering out-of-hospital cardiac arrest receive any bystander cardiopulmonary resuscitation (CPR). Bystander CPR is associated with improved odds for survival. The PulsePoint smartphone application alerts users in the vicinity of a cardiac arrest to facilitate immediate citizen bystander resuscitation. Addressing implementation barriers may provide an opportunity to increase effectiveness of the application. PulsePoint is currently active in over 400 communities in the United States. Our objective was to identify modifiable barriers to optimal implementation of the PulsePoint smartphone application.

## Methods

We conducted a structured survey delivered via Survey Monkey optimized for mobile devices. All alerted PulsePoint users between 28 June 2012 and 1 November 2013 were sent an invitation to participate in the survey. Survey responses associated with test activations and emergency medical services (EMS) professionals on active duty were excluded from the analysis.

## Results

Of 4,827 survey requests sent, 995 responded and completed our survey (response rate 21%). Respondents identified themselves as firefighters (30%), paramedics (19%), EMTs (16%), nurses (4.4%), MDs (0.83%) and other professions (50%). Of those who received a PulsePoint alert, 23% (157/690) responded by making their way towards the location of the emergency. Of those who responded, 70% (110/157) arrived on scene. Most of those who did not arrive said they saw professional rescuers already on scene and turned back (52%, 24/47). Of those who arrived on scene, only 34% (37/110) found a person unconscious and not breathing normally. The majority found a person on scene who was not suffering cardiac arrest. Of those who arrived on scene prior to EMS and also found a cardiac arrest victim on scene who required resuscitation, 80% (8/10) performed bystander CPR.

## Conclusion

We observed a very high proportion of bystander CPR (80%) for victims of out-of-hospital cardiac arrest when PulsePoint users arrived before EMS. This suggests that optimized PulsePoint implementation may increase community bystander CPR rates. Alert specificity for cardiac arrest may be too low (the cry wolf scenario) with current default activation criteria. Also, the current activation radius (0.5 mile) may be too large because EMS frequently arrive before the PulsePoint responder.

